# An Exploration of Patient Navigation and Community Health Worker Activities Across National Comprehensive Cancer Control Programs

**DOI:** 10.1089/heq.2018.0053

**Published:** 2018-12-14

**Authors:** Elizabeth A. Rohan, Renee McDougall, Julie S. Townsend

**Affiliations:** ^1^ Division of Cancer Prevention and Control, Centers for Disease Control and Prevention, Atlanta, Georgia.; ^2^ Surgery Department, Animal Medical Center, Manhattan, New York.

**Keywords:** community health workers, comprehensive cancer control, disparities, patient navigation

## Abstract

**Purpose:** Health disparities persist across the cancer care continuum. Patient navigator (PN) and community health worker (CHW) interventions are designed to increase health equity. National Comprehensive Cancer Control Program (NCCCP) awardees develop and implement plans to coordinate cancer prevention and control activities, including supporting PN and CHW interventions. This content analysis examined NCCCP action plans to assess the extent to which jurisdictions report engaging in PN and/or CHW activities.

**Methods:** We abstracted PN and CHW content from NCCCP action plans and coded content according to specific areas of PN and/or CHW intervention (e.g., screening, survivorship, and cancer type), used descriptive statistics to characterize overall results, and calculated chi-squares to determine whether programs engaged PNs and CHWs differently.

**Results:** Eighty-two percent (*n*=53) of 65 NCCCP action plans had content related to PN and/or CHW activities, with more PN language (83%) than CHW (58%). These action plans described engaging PNs and CHWs in activities across the cancer continuum, but particularly for screening (60%) and survivorship (55%). Eighty-one percent of these plans described activities related to workforce development, such as training and standardizing roles and competencies. Programs engaged CHWs more often than PNs for outreach and in community settings.

**Conclusion:** The majority of NCCCP awardees reported engaging in PN and/or CHW activities. Understanding how NCCCP awardees engage PNs and CHWs, including awardees' needs for workforce development in this area, can help Centers for Disease Control and Prevention provide more focused technical assistance as programs increase engagement of PNs and CHWs to improve health equity.

## Introduction

Racial and ethnic minorities, rural communities, and those with low socioeconomic status, including those who are un- or under-insured, bear a disproportionately high burden of cancer in the United States.^1–4^ These disparities persist across the cancer continuum, from prevention to screening, treatment, and long-term survivorship.^4–6^ Barriers to cancer prevention, screening, and care often include fear or distrust of the medical community, lack of or inadequate health insurance coverage, financial hardship, low levels of health literacy and education, and logistical barriers, such as lack of transportation.^7,8^ The social determinants of health, that is, the social, economic, cultural, and physical contexts within which people live, are considered important when addressing health disparities.^9–11^

Patient navigation (PN) and community health worker (CHW) interventions have been demonstrated to reduce health disparities by addressing the social determinants of health.^9,12–18^ Cancer PN is a process that provides “individualized assistance to cancer patients, families, and caregivers to help overcome healthcare system barriers and facilitate timely access to quality health and psychosocial care from pre-diagnosis through all phases of the cancer experience.”^19^ PNs assist patients in overcoming barriers to cancer screening and treatment, offer peer counseling, provide linkages to financial and community resources, and provide culturally competent patient education, particularly among (but not limited to) the underserved.^20^ Studies have shown the effectiveness of PN in increasing patient completion of cancer screening and reducing disparities among the medically underserved.^12,13,21,22^ A recent systematic review of both PN and CHW interventions at Federally Qualified Health Centers, which provide primary care to medically underserved communities, found that PN/CHW interventions were effective in increasing cancer screening rates.^9^ Similarly, a systematic review of CHW interventions and mammography found that such interventions improve mammography rates.^17^

Dr. Harold Freeman pioneered PN in 1990 to address the challenges low-income, medically underserved populations experience with access to timely cancer screenings, treatment, and care.^13^ PNs often come from the community they serve, but they typically function in clinical settings to reduce barriers to cancer screening and timely treatment, act as liaison to the medical team, and help cancer patients and families manage the logistics of cancer treatment, which often includes various specialists and time-intensive treatment. Depending on the needs of the health care setting, PNs may be lay members of the community they serve or health care professionals (usually nurses or social workers).^23^ Their scopes of practice are reflective of their backgrounds.^24^

CHW programs gained popularity in the United States in the 1960s, but their international origins date back to the 19th century.^15^ CHWs are frontline public health workers who are trusted members of the community they serve and provide the cultural expertise to help link the community to health and social services.^9,15,25–27^ Depending on community or context, CHWs are also known as community health advisors, outreach workers, community health representatives (CHRs), promotoras(es), peer counselors, lay health advocates, and peer health educators, among other titles.^28,29^ Historically, CHWs have worked through community-based organizations focusing on health education, prevention, and screening, while PNs have functioned in clinical settings to navigate cancer patients through the health care system,^23^ although recent trends using CHWs within health care teams and navigators for outreach have blurred those boundaries.^30–32^

PNs and CHWs have become increasingly valued members of the U.S. health care workforce in addressing health disparities and other public health priorities, particularly following changes in health care payment structures and grant funding under the Patient Protection and Affordable Care Act,^33,34^ in addition to recommendations and guidance contained in the U.S. Department of Health and Human Services' National Prevention Strategy.^35^ Since 2015, the American College of Surgeon's Commission on Cancer has required PN services as a component of accreditation.^13^

The Centers for Disease Control and Prevention (CDC) funds the National Comprehensive Cancer Control Program (NCCCP) in all 50 states, the District of Columbia, and several tribes, tribal organizations, U.S. territories, and Pacific Island Jurisdictions to broadly promote cancer prevention and control activities and interventions.^36^ NCCCP awardees focus their activities on the following priority areas: emphasizing primary prevention of cancer; assisting with coordination of early detection and treatment activities; addressing public health needs of cancer survivors; helping with using policy, systems, and environmental changes (PSEs) to guide sustainable cancer control; and promoting health equity and reducing disparities.^37^ Each NCCCP awardee works with a coalition and other stakeholders to develop and implement comprehensive cancer plans, which are blueprints for addressing their population's particular cancer burden, and they document progress in yearly action plans.^38^ Since PNs and CHWs could be used to address most of the NCCCP priority areas, we sought to examine the extent to which awardees are engaging in activities related to and supportive of PN and/or CHW interventions. The objective of this content analysis was to examine NCCCP action plans for incorporation of PN and/or CHW interventions in cancer control.

## Methods

We abstracted content from NCCCP action plans (*n*=65) in December 2015 from CDC's Chronic Disease Management Information System (CDMIS; www.cdc.gov/cdmis/index.html) using these keywords: navigate, navigator(s), navigation, peer educator, community navigator, community health aid, lay health worker, Promotora(s), peer counselor, peer leader, lay health educator, CHWs, PNs, CHRs, lay health advisor, peer health advisor, peer health educator, community health advisor, and outreach worker. The NCCCP action plans spanned a 3-year time period, beginning in 2012 at the onset of a new 5-year funding cycle (2012–2017). As part of funding expectations, NCCCP awardees submit via CDMIS annual action plans that outline specific objectives and activities to be accomplished in the upcoming year, and report on progress they made during the current project year for each action plan objective. CDMIS meets all federal requirements and approvals for public data collection (OMB No. 0920-0841).

After abstraction, we deleted duplicate text entries and reviewed action plan text for inclusion, based on where in the action plan the term appeared. Only plans that used the keyword in text as part of an objective, strategy, or goal were included. For example, we excluded content describing webinars attended by NCCCP awardees on PN/CHW topics or descriptions of coalition members who were PNs or CHWs, without reference to a planned activity or intervention engaging PNs or CHWs or involving workforce development.

Beginning with a summative approach to content analysis^39^ and later using an inductive approach we (E.A.R. and R.M.) open-coded text, according to specific areas of PN and/or CHW intervention (e.g., screening, survivorship, and cancer type). We (E.A.R., R.M., and J.T.) developed a detailed codebook that defined codes and specified how to apply them to text. One researcher (R.M.) had the primary responsibility of coding content according to codebook rules, using NVivo10^®^ (Victoria, Australia) to manage the data, but had frequent meetings with other investigators (E.A.R. and J.T.) to review analysis. Analysis first assessed whether action plans included PN and/or CHW language. For those action plans that did contain PN and/or CHW language, we coded to reflect the context in which the PN and/or CHW language was used. We used descriptive statistics to characterize overall results and calculated chi-squares to determine differences in PN and/or CHW activities and settings among programs.

## Results

**FIG. 1.**
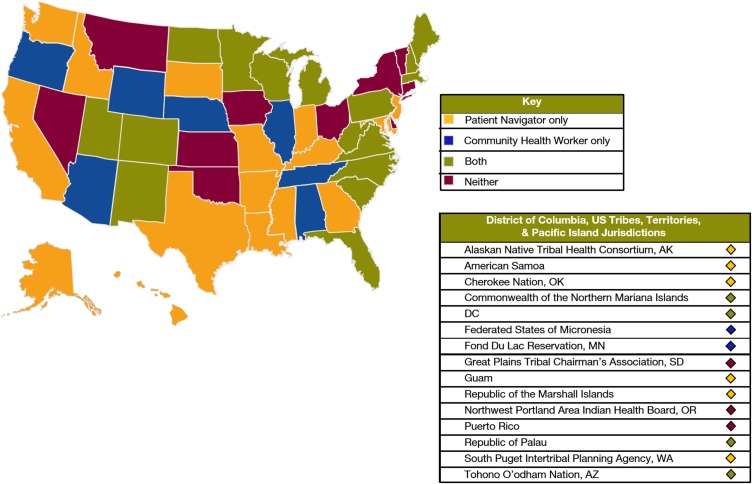
U.S. map depicting states with action plans using patient navigator and/or community health worker terms.

Figure 1 depicts whether action plans from each NCCCP awardee contained PN content only, CHW content only, both, or neither. Twenty-three (35%) of plans contained PN language only, 9 (14%) contained CHW language only, 20 (31%) contained PN and CHW content, and 13 plans (20%) had no language related to PN or CHW. A total of 82% (*n*=53) of 65 NCCCP action plans had content related to PN and/or CHW activities. Of those 53 with PN and/or CHW language, more action plans contained PN (83%; *n*=44) than CHW (58%; *n*=31) language. Among plans that contained either PN or CHW language, this pattern held true in that 22 mentioned only PN (42%) and 9 mentioned only CHW (17%) language.

### Cancer type and cancer continuum

**FIG. 2.**
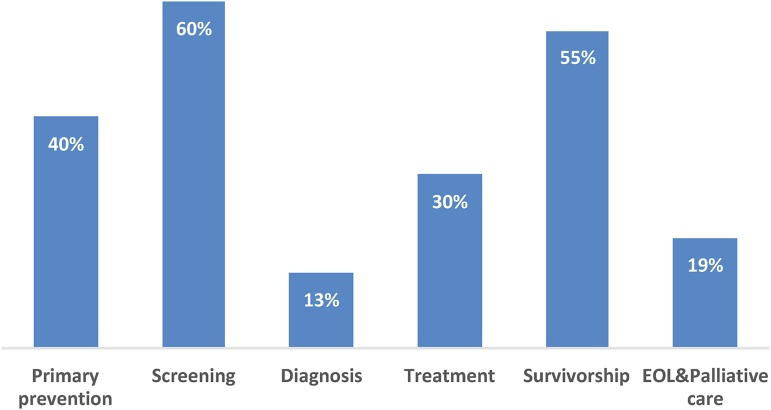
Percentage of action plans with PN and/or CHW language by cancer continuum area (*n*=53). Note: Percentages do not total 100% because action plans may have specified more than one area across the cancer continuum. CHW, community health worker; EOL, end-of-life; PN, patient navigation; PSE, policy, systems, and environmental change.

While 36% (*n*=19) of action plans with PN and/or CHW language did not specify which type(s) of cancers PNs/CHWs would work to prevent or control (25%; *n*=13), those that did most commonly cited breast (47%; *n*=25), cervical (45%; *n*=24), and colorectal (40%; *n*=21) cancers (Table 1). Additionally, 32% of action plans described PN and/or CHW language in relation to chronic diseases in addition to cancer. Furthermore, action plans specified intervening at various points across the cancer continuum (Fig. 2), but primarily with cancer screening (60%; *n*=32) and survivorship (55%; *n*=29). Examples of interventions include promoting cancer screening by providing tailored education and arranging transportation services and ensuring survivors are aware of and have access to survivorship resources and participating in survivorship committees at cancer centers.

**Table 1.** **Percentage of Action Plans with Patient Navigation and/or Community Health Worker Language by Type of Cancer (*n*=53)**

Type of cancer or chronic disease	*n* (%)
Cancer not specified	19 (36)
Breast	25 (47)
Cervical	24 (45)
Colorectal	21 (40)
Lung	3 (6)
Prostate	3 (6)
Chronic disease (including cancer)	17 (32)

Note: Percentages do not total 100% because action plans may have specified more than one cancer type.

### PN/CHW patient-level and systems-level activities

**FIG. 3.**
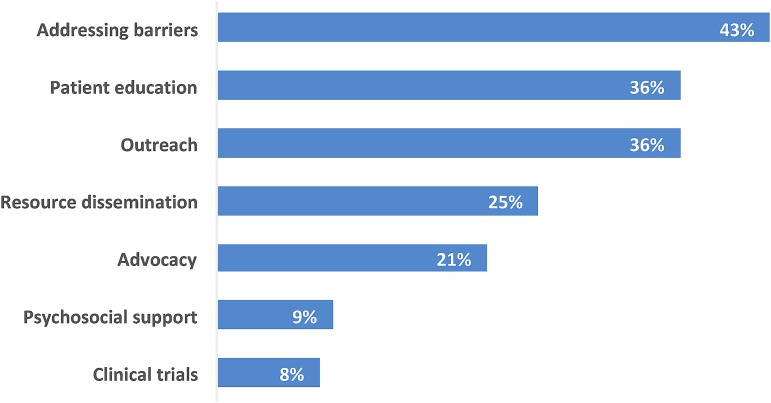
Percentage of action plans with PN and/or CHW language related to individual patient-level activities (*n*=53). Note: Percentages do not total 100% because action plans may have specified more than one activity.

**FIG. 4.**
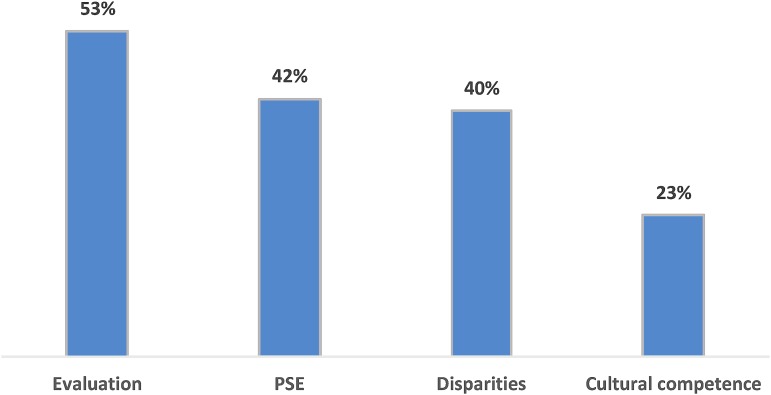
Percentage of action plans with PNs and/or CHW language related to systems-level activities (*n*=53). PSE, policy, systems, and environmental change.

Within action plans with PN and/or CHW language, PNs/CHWs were most often involved in these patient-level activities: addressing barriers (43%; *n*=23), patient education (36%; *n*=19), outreach (36%; *n*=19), resource dissemination (25%; *n*=13), and advocacy (21%; *n*=11; Fig. 3). They were relatively less frequently providing psychosocial support (9%; *n*=5), or discussing clinical trials (8%; *n*=4). PNs/CHWs also were linked with systems-level activities (Fig. 4), such as evaluation (53%; *n*=28); PSE (42%; *n*=22); reducing disparities (40%; *n*=21); and cultural competence (23%; *n*=12). Table 2 shows examples of PN and/or CHW language from action plans, along with corresponding codes, related to both patient-level and systems-level activities.

**Table 2.** **Examples of Patient Navigation and/or Community Health Worker Activities in Action Plans**

PN/CHW activity or area	Action plan examples
Addressing barriers	[To reduce the need for palliative care patients to travel long distances], the Cancer Program [will] pilot…our long-distance palliative care appointment system. The oncologist will work with community health workers [who will be at the patient's home and] order necessary tests, [talk] with family members about daily care, and respond to the cares and concerns of the patient.
	Utilizing the promotora model, staff provides education on the importance of breast and cervical cancer screenings throughout the community and help women in underserved populations access free or low-cost services…Patient navigators helped women access screening programs by helping them complete necessary paperwork, finding transportation to screening appointments (including carpools), and providing translation services when needed. They also provided reminder phone calls to women with screening appointments…and provided culturally sensitive breast health education and navigation services.
Cultural competence	This activity is focused on outreach implemented by the Cancer Program Outreach Worker to [name of community] women through Circle Of Life: American Cancer Society's Cancer Education Program developed specifically for American Indians…The Circle of Life program provides education and program promotion materials that are culturally appropriated for the service population…The new program is web-based and has the capacity to make it culturally specific to an individual tribal community through artwork and other graphic images and Tribal Health Services information.
	Increase the number of Community Health Workers trained in end-of-life issues. The end-of-life training is intended to increase the skills of community health workers and allied health workers to discuss advance care planning, hospice care, palliative care, and other end-of-life issues with their clients. A culturally sensitive end-of-life training curriculum has been developed, and we are communicating with our community partner about hosting at least one training before [the end of the funding period].
Disparities	Conducting targeted outreach to increase the rate of mammography screening among groups that experience high mortality rates from breast cancer will ensure screenings are provided to those who are disproportionately impacted by the disease…[We will] conduct “Cancer in Your Community” training for community health workers and allied health workers on cancer early detection…Through lecture and interactive teaching strategies, participants learn about cancer and cancer screening, develop skills to teach others about cancer and screening, and learn about cancer resources in their communities.
	[The Health Department] contracted with four local communities already implementing chronic disease prevention through policy and systems change to integrate primary cancer prevention into their current efforts…grantees are in four, low-income, diverse cities in [our area]…all four grantees incorporated information about the link between obesity and cancer into their training for Community Health Workers (CHW), patient navigators, and Volunteer Health Advisors (VHA). [The Health Department also] funds 3 community-based organizations in high-need areas of the state to provide education, patient navigation and linkages to clinical and social support services with the goal of decreasing colorectal, breast, and cervical cancer screening disparities.
PSE	Increase the percent of subject matter expert staff time designated for implementation of PSE efforts in survivorship for tribal health systems improvement…In addition to PSE involving an increased role for village Behavioral Health Aides in caring for cancer survivors, the Cancer Program has also been involved with a statewide effort to increase the presence of patient navigators in facilities and as champions in communities.
	Increase the number of funding models proposed to PN/CHW Collaborative…To that end [the health department] co-sponsored a Patient Navigation and Community Health Work Sustainability Summit….The intent of this gathering was to engage in critical dialog with key stakeholders, health systems representatives, and policy-makers. Participants evaluated successful [state] and national models and developed recommendations for [the state's] sustainable model for patient navigators and community health workers…Based on analysis, develop and share a funding model proposal with the CHW/PN Collaborative.
Workforce	Training: Increase the number of Community Health Workers (CHWs) trained in standard core competencies to increase use of clinical screenings and link community clinical preventive services. Support development of a statewide system to train CHWs in core competences.
	Building capacity: Increase the number of patient navigation systems…to improve quality of life among cancer survivors…hire and train a part-time HOPE cancer navigator to staff the HOPE Center Resource Room…The HOPE cancer navigator will be available to assist anyone affected by cancer, including family and caregivers.

CHW, community health worker; PN, patient navigation; PSE, policy, systems, and environmental change.

### Workforce development

Most (81%; *n*=43) of the action plans with PN and/or CHW language reported their programs were engaged in developing the PN and/or CHW workforce (Table 3). The majority (88%; *n*=38) of these described engaging in training of PNs and/or CHWs, and over half (58%; *n*=25) mentioned work related to building PN and/or CHW capacity within their jurisdiction. Other workforce development activities reported in the action plans related to financing (35%; *n*=15), networking (35%; *n*=15), and standardizing either by working toward uniformity in definitions, roles, and core competencies for PNs and/or CHWs (21%; *n*=9), or by credentialing (16%; *n*=7).

**Table 3.** **Patient Navigation/Community Health Worker Workforce Development Categories Within National Comprehensive Cancer Control Program Action Plans (*n*=43)**

Workforce development category	*n* (%)
Training	38 (88)
Building capacity	25 (58)
Financing	15 (35)
Networking	15 (35)
Standardization/uniformity	9 (21)
Standardization/credentialing	7 (16)

### PN/CHW role differentiation

Table 4 illustrates that the majority of action plan language showed no statistical differences in role differentiation between PNs or CHWs; however, there are some exceptions. Action plan language more often described engaging CHWs in outreach, patient education, cultural competence, community settings, and primary prevention than PNs. Action plan language more often described engaging PNs, rather than CHWs, in relation to cancer survivorship.

**Table 4.** **Action Plan Language Associated with Community Health Workers Versus Patient Navigators**

	Role	
	CHW (*n*=88), *n* (%)	PN (*n*=107), n (%)
Patient-level activities		
Resource dissemination	18 (21)	13 (12)
Outreach^*^	31 (35)	15 (14)
Patient education^*^	28 (32)	19 (18)
Addressing barriers	26 (30)	24 (22)
Systems-level activities		
Cultural competence^*^	15 (17)	7 (7)
Reducing disparities	21 (24)	16 (15)
PSE	22 (25)	26 (24)
Evaluation	31 (35)	32 (30)
Workforce development		
Building capacity	23 (26)	25 (23)
Financing	14 (16)	17 (16)
Networking	14 (16)	20 (19)
Training	41 (47)	55 (51)
Setting		
Community^*^	32 (36)	9 (8)
Clinic	13 (15)	27 (25)
Cancer continuum		
Primary prevention^*^	28 (32)	17 (16)
Screening	36 (41)	33 (31)
Survivorship^*^	16 (18)	45 (42)

^*^ Indicates significant difference, *p*<0.05.

## Discussion

This is the first assessment of PN/CHW content in NCCCP action plans. The vast majority of NCCCP awardees reported engaging in PN and/or CHW activities, indicating a prioritization of this intervention in cancer prevention and control consistent with cancer PN literature.^40^ Since PN originated within cancer,^20^ it is not surprising that our review showed that this term was more prevalent than CHW in NCCCP action plans.

Our findings that NCCCP action plans often described engaging PNs/CHWs in screening and in relation to breast, cervical, or colorectal cancer are also supported by the literature. A systematic review found that most PN efficacy studies have been conducted in relation to screening for breast, cervical, or colorectal cancers.^40^ Action plans described PNs and CHWs most often in relation to addressing barriers and providing patient education, which is also consistent with the literature.^8^ Additional evidence is needed for assessing the efficacy of engaging PNs and CHWs in screening for lung cancer or increasing uptake of HPV vaccination. Since lung cancer screening is complex^41,42^ and HPV vaccination rates remain low in the United States,^43,44^ there may be a role for PNs and or CHWs in one or both of these arenas as well.

Workforce development is an important aspect in advancing the work of PNs and CHWs.^45–48^ NCCCP action plans reflected the many ways programs are working to develop and build capacity within PN and CHW workforces by providing trainings and networking opportunities, working to secure solid sources of financing, working to standardize roles and competencies, and working toward credentialing. Not every NCCCP awardee reported being involved in each of these workforce development activities, but it is clear that many awardees have prioritized taking part in PN and/or CHW workforce development to increase the capacity of PNs and CHWs to serve populations within their jurisdictions.

Finally, our findings showed that action plans used CHW language more often related to outreach, patient education, cultural competence, community settings, and primary prevention while action plans used PN language more often related to cancer survivorship. This is consistent with the established perception that CHWs are trusted members of the communities they serve.^29,45,46,49^ PN was pioneered in the field of oncology^20^; the association of PNs with individuals post-diagnosis (cancer survivors) seems consistent with this history.

### Limitations

While providing detailed information about PN and/or CHW activities within NCCCP, action plans do not reflect all that is being done in cancer by PNs and/or CHWs. Specific information may not be contained in NCCCP action plans, either for timing reasons (only 3 years of action plans were examined during the 5-year funding cycle) or reporting reasons (an NCCCP awardee may not report all PN/CHW-related activities they are engaged in, especially if financial resources for the activities are shared across health department entities or programs).^45^

Even though action plans are semi-structured, each one varies in terms of the depth and relevancy of information provided. Therefore, we may not have captured all activities or have enough detail to adequately capture the focus of the activity. Although unlikely, since most NCCCPs had PNs and/or CHW content, we could have missed retrieving content that used an alternative abbreviation for PN or CHWs. These data are also self-reported and potentially could be subject to social desirability bias. Finally, NCCCP awardees did not always specify in their action plans which cancer populations their PN and/or CHW efforts were focused on; this created difficulty in assessing the specific nature of this work in increasing health equity in underserved populations.

### Strengths

As noted, this is the first study to quantify PN and CHW activity in cancer across the NCCCP and can be used as a baseline in future analyses. Additionally, the coding of action plan content followed a rigorous process that involved all 3 researchers to minimize bias to the greatest extent possible in a content analysis.

## Conclusion

Understanding how NCCCP awardees engage PNs and CHWs, including awardees' needs for workforce development in this area, can help CDC provide more focused technical assistance to NCCCP awardees as they work to support cancer prevention and screening, improve the quality of life among cancer survivors, and help reduce disparities in cancer prevention and control. This review can serve as a baseline assessment of PN/CHW activities in CCC plans and give some indication as to the integration of these approaches into comprehensive cancer control efforts. Future PN/CHW efforts among NCCCP awardees can include the specific population addressed, which may allow for easier assessment of the effects of PNs/CHWs in reducing health disparities. Reporting on the specific population served may additionally provide best practices for other NCCCP awardees working within similar populations. Overall, our findings highlight similarities and distinguishing characteristics among how the NCCCP awardees engage PNs and CHWs and can help inform the implementation and evaluation of cancer prevention and control efforts within both public health and oncology health care programs that engage PNs and CHWs. Ongoing support of the implementation of PN and CHW interventions and continued assessment of their added value in cancer control offers public health programs, including the NCCCP, a means to further improve chronic disease health equity.^9^

## Abbreviations Used

CDC: Centers for Disease Control and Prevention
CDMIS: Chronic Disease Management Information System
CHRs: community health representatives
CHW: community health worker
NCCCP: National Comprehensive Cancer Control Program
PN: patient navigation
PSE: policy, systems, and environmental change
